# Voxel‐wise partial volume correction method for accurate estimation of tissue sodium concentration in ^23^Na‐MRI at 7 T

**DOI:** 10.1002/nbm.4448

**Published:** 2020-12-03

**Authors:** Sang‐Young Kim, Junghyun Song, Jong‐Hyun Yoon, Kyoung‐Nam Kim, Jun‐Young Chung, Young Noh

**Affiliations:** ^1^ Neuroscience Research Institute Gachon University Incheon Republic of Korea; ^2^ Department of Biomedical Engineering Gachon University Incheon Republic of Korea; ^3^ Department of Neuroscience Gachon University College of Medicine Incheon Republic of Korea; ^4^ Department of Neurology, Gil Medical Center Gachon University College of Medicin e Incheon Republic of Korea

**Keywords:** ^23^Na‐MRI, 3D kernel, linear regression (LR), modified least trimmed square (mLTS), partial volume correction (PVC), spatial blurring, tissue sodium concentration (TSC)

## Abstract

Sodium is crucial for the maintenance of cell physiology, and its regulation of the sodium‐potassium pump has implications for various neurological conditions. The distribution of sodium concentrations in tissue can be quantitatively evaluated by means of sodium MRI (^23^Na‐MRI). Despite its usefulness in diagnosing particular disease conditions, tissue sodium concentration (TSC) estimated from ^23^Na‐MRI can be strongly biased by partial volume effects (PVEs) that are induced by broad point spread functions (PSFs) as well as tissue fraction effects. In this work, we aimed to propose a robust voxel‐wise partial volume correction (PVC) method for ^23^Na‐MRI. The method is based on a linear regression (LR) approach to correct for tissue fraction effects, but it utilizes a 3D kernel combined with a modified least trimmed square (3D‐mLTS) method in order to minimize regression‐induced inherent smoothing effects. We acquired ^23^Na‐MRI data with conventional Cartesian sampling at 7 T, and spill‐over effects due to the PSF were considered prior to correcting for tissue fraction effects using 3D‐mLTS. In the simulation, we found that the TSCs of gray matter (GM) and white matter (WM) were underestimated by 20% and 11% respectively without correcting tissue fraction effects, but the differences between ground truth and PVE‐corrected data after the PVC using the 3D‐mLTS method were only approximately 0.6% and 0.4% for GM and WM, respectively. The capability of the 3D‐mLTS method was further demonstrated with in vivo ^23^Na‐MRI data, showing significantly lower regression errors (ie root mean squared error) as compared with conventional LR methods (*p* < 0.001). The results of simulation and in vivo experiments revealed that 3D‐mLTS is superior for determining under‐ or overestimated TSCs while preserving anatomical details. This suggests that the 3D‐mLTS method is well suited for the accurate determination of TSC, especially in small focal lesions associated with pathological conditions.

## Graphical abstract

A voxel‐based partial volume correction method in ^23^Na‐MRI is proposed in order to reduce spatial blurring effects as well as for accurate determination of tissue sodium concentration (TSC). The method is validated with simulation, showing that the performance of a 3D kernel combined with a modified least trimmed square (3D‐mLTS) method is the best for accurate determination of the TSC with preservation of detailed anatomical information, as compared with a conventional 2D or 3D linear regression (LR) approach.

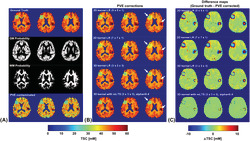

**Abbreviations**
^1^H‐MRI: proton MRI
^23^Na‐MRI: sodium MRI
ANLM: adaptive non‐local mean
ANOVA: analysis of variance
ASL: arterial spin labeling
CSF: cerebral spinal fluid
FOV: field of view
FWHM: full width at half maximum
GM: gray matter
GTM: geometric transfer matrix
LR: linear regression
mLTS: modified least trimmed square
Na^+^/K^+^‐ATPase: sodium‐potassium pump
PET: positron emission tomography
PSF: point spread function
PVC: partial volume correction
PVE: partial volume effect
RMSE: root of the mean squared error
SNR: signal‐to‐noise ratio
T1w: *T*_1_‐weighted
TPM: tissue probability map
TSC: tissue sodium concentration
WM: white matter

## 1 INTRODUCTION

With recent advances in MRI hardware (eg high‐field magnets and strong gradient capabilities), sodium MRI (^23^Na‐MRI) has received increasing attention as it provides direct biochemical information on tissue viability such as cell membrane integrity.^1^, ^2^ Sodium is a crucial component that helps to maintain cell physiology via the sodium‐potassium pump (Na^+^/K^+^‐ATPase), which is a high energy consumer and dependent upon adenosine triphosphate production.^3^ Abnormal cellular energy production or dysregulation of Na^+^/K^+^‐ATPase can induce imbalances between intracellular and extracellular Na^+^ concentrations. A loss of Na^+^ homeostasis induces an increase in intracellular Na^+^ concentrations.^4^ There has been significant clinical interest in the use of ^23^Na‐MRI to detect early physiological changes in various neurological diseases, including Alzheimer's disease,^5^ Huntington's disease,^6^ stroke,^7^ multiple sclerosis,^8^, ^9^, ^10^ and brain tumors.^11^

A major obstacle in the clinical application of ^23^Na‐MRI is its relatively low MR sensitivity when compared with conventional proton MRI (^1^H‐MRI), despite yielding the second strongest NMR signal among all nuclei present in biological tissue. This is due to low tissue sodium concentrations (TSCs) (around 40 mM) in the brain and biexponential *T*_2_ relaxation behavior with a very short *T*_2_.^12^ TSC is defined as a weighted average of intracellular sodium (approximately 10‐15 mM) and extracellular sodium (approximately 140 mM) concentrations,^9^ which consequently leads to a TSC of approximately 40 mM in brain tissue. Due to the low MR sensitivity, sufficient averaging or large voxel sizes (typically 3‐5 mm) are required to increase the signal‐to‐noise ratio (SNR) in ^23^Na‐MRI. However, the resultant low spatial resolution introduces partial volume effects (PVEs) into the estimated TSC. It is therefore necessary to correct for PVEs to avoid under‐ or overestimation of TSC in the brain.

The limited resolution of ^23^Na‐MRI leads to “tissue fraction” effects, reflecting underlying tissue heterogeneity, as well as “spill‐over” effects between regions. Specifically, the tissue fraction effect refers to intra‐voxel partial volumes (ie a single voxel containing more than one tissue type), whereas the spill‐over effect refers to signal leakage between adjacent voxels due to a point spread function (PSF). Correcting for tissue fraction effects in general requires the tissue segmentation of high‐resolution MRI data, and the spill‐over can be accounted for with a knowledge of PSF. To address the latter problem, several partial volume correction (PVC) algorithms have been proposed for positron emission tomography (PET),^13^, ^14^, ^15^ while the former problem has been addressed by arterial spin labeling (ASL) MRI.^16^, ^17^ Thus, complete PVC ideally involves a combination of corrections for both the spill‐over and tissue fraction effects.

A simple but powerful PVC method that corrects for tissue fraction effects is a linear regression (LR) algorithm based on a model that represents the voxel intensity as a weighted sum of pure tissue contribution, where the weighting coefficients are each tissue's fractional volume in the voxel.^16^ In the LR method, the algorithm tries to solve the underdetermined system (ie, for a single voxel, there are three unknowns in one equation available; details in Section 2) by utilizing the signals from neighboring voxels (eg kernel). Thus, one needs to assume that the estimated parameters over the regression kernel remain constant, thereby inducing inevitable smoothing effects in the PVE‐corrected image. It is worth noting that, as the smoothing effects caused by the regression method depend on the detailed structure of the local tissue fractions, they are different from spatial blurring induced by smoothing filters. On the other hand, a representative method for correcting spill‐over effects is to use a geometric transfer matrix (GTM), which incorporates anatomical information to correct across the regions that are assumed to be homogeneous. Although the GTM method is straightforward to implement and remains the most widely used PVC technique, there is a limitation that it does not provide a PVE‐corrected image, as the correction is performed at a regional level.

In conventional MRI with a Cartesian readout, it has been commonly assumed that the effect of PSF is negligible due to a relatively narrow full width at half maximum (FWHM) of the PSF. However, most ^23^Na‐MRI studies have been conducted with a dedicated pulse sequence with a 3D radial acquisition scheme, resulting in a larger FWHM of the PSF, which consequently leads to signal spill‐over across neighboring voxels. In this aspect, we minimize PSF‐induced signal spill‐over by acquiring sodium data using a Cartesian readout and further reduce the spill‐over effect by taking the PSF into account in this work.

The primary objective for this work is to correct for tissue fraction effects in the accurate estimation of TSC in the brain, but the spill‐over effects due to the PSF are also considered for the PVC in ^23^Na‐MRI. Thus, we propose a novel voxel‐wise PVC method accounting for both tissue fraction and spill‐over effects in ^23^Na‐MRI. The proposed method still employs a kernel‐based regression approach, but seeks to minimize inherent spatial blurring effects by using a 3D kernel combined with modified least trimmed squares (3D‐mLTS).^17^, ^18^ To validate the method, Monte Carlo simulations were performed by generating a pseudo‐sodium MR image. Optimized parameters (ie kernel size, trimming parameter) for the 3D‐mLTS method are applied on an in vivo ^23^Na‐MRI dataset acquired with 7 T MRI. We further examined the extent of signal spill‐over in a phantom experiment with a Cartesian acquisition scheme. Finally, individual in vivo TSC maps were calculated using a ventricle signal as a reference, while the contributions of high TSC in cerebral spinal fluid (CSF) to gray matter (GM) and/or white matter (WM) due to the PSF were considered for accurate TSC estimation.

## 2 MATERIALS AND METHODS

### 2.1 Theory

**FIGURE 1:**
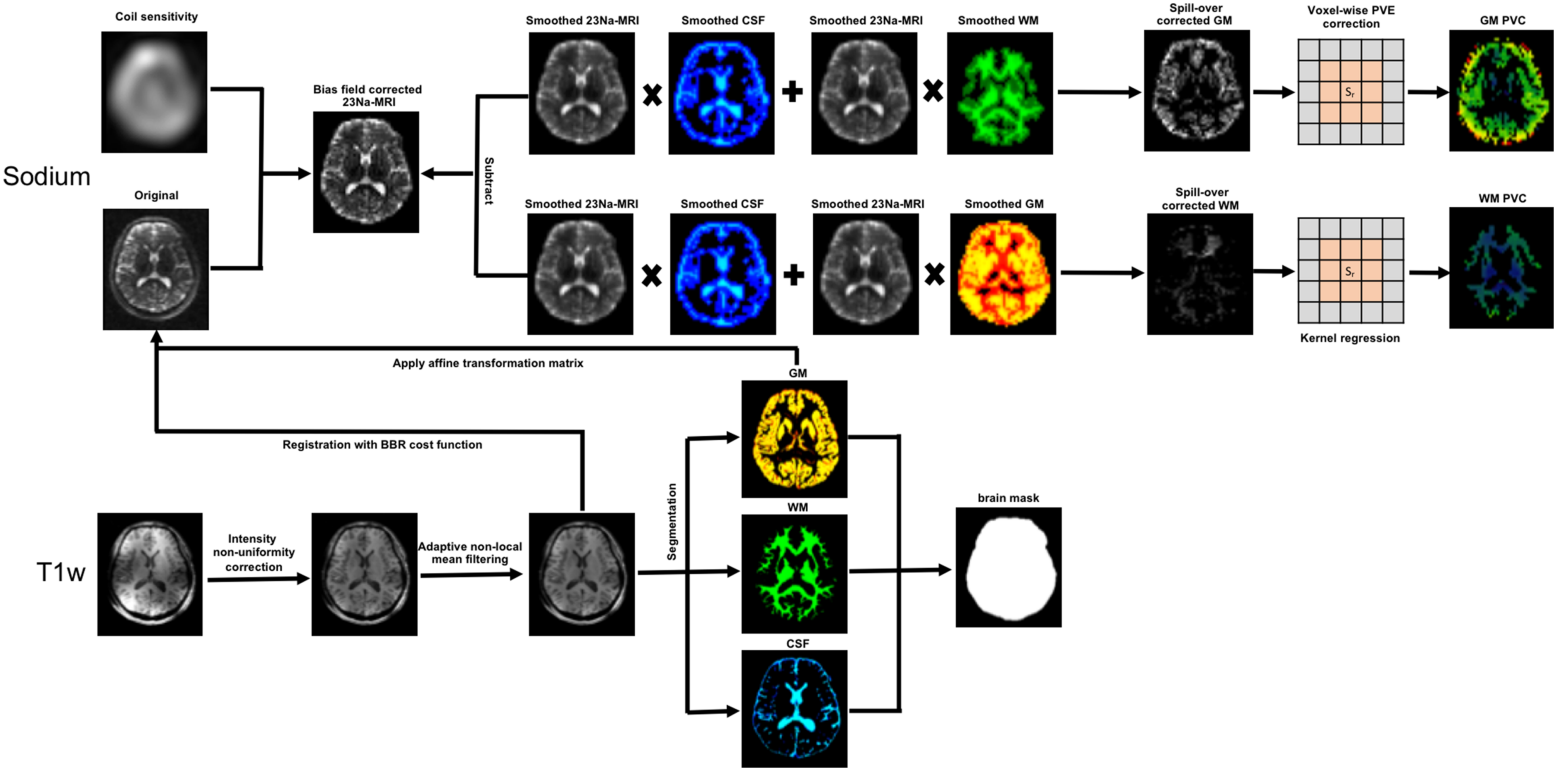
Outline of the processing method employed for PVC in ^23^Na‐MRI. Prior to applying PVC to the images, the bias field was corrected for using a coil sensitivity map followed by correction for spill‐over effects using a Gaussian PSF with an FWHM of 4.8 mm. The tissue fraction effects, which require high‐resolution anatomical images segmented into GM, WM, and CSF, are then corrected by kernel regression. Details of the 3D‐mLTS method can be found in Section 2

Given the limited spatial resolution of ^23^Na‐MRI, the signal in each voxel contains contributions from GM, WM, and CSF. Ideally, these contributions should be separately delineated. The signal intensity in a voxel of ^23^Na‐MRI can be described with the following relationship:

(1) $$S_{r}=P_{\mathrm{GM},r}M_{\mathrm{GM},r}+P_{\mathrm{WM},r}M_{\mathrm{WM},r}+P_{\mathrm{CSF},r}M_{\mathrm{CSF},r}$$

where *S*_*r*_ is the measured signal intensity at voxel location *r*, and *P*_GM,*r*_, *P*_WM,*r*_, and *P*_CSF*,r*_ are the probabilities of GM, WM, and CSF at voxel location *r*, respectively. A tissue probability map (TPM) can be obtained from the segmentation of a high‐resolution *T*_1_‐weighted (T1w) MR image. The objective is to estimate the pure signal intensities of each component (*M*_GM,*r*_, *M*_WM,*r*_, *M*_CSF,*r*_), which are the three unknown parameters in Equation 1. It is not possible to solve for three unknowns considering that there is only one equation available. By assuming that the TSC is constant over the kernel, more equations can be considered where the regression approach is used. The solution can be found by converting Equation 1 into matrix form:

(2) $$\bar{m}={\left(P^{T}\cdot P\right)}^{-1}\cdot P^{T}\cdot \bar{S\;}$$

where $\bar{S}$ is the column vector of observed signals at each voxel in the regression kernel, superscript T denotes the matrix transpose, (*P*^T^ · *P*)^−1^ · *P*^T^ is the pseudoinverse of *P*, and $\bar{m}$ represents the pure tissue signal intensities for GM, WM, and CSF. It should be noted that the TPM matrix over the kernel should not be rank deficient, which limits the use of kernels of smaller sizes (eg 3 × 3 kernel). As the regression depends on (*P*^T^ · *P*)^−1^ being non‐singular, for any situations in which no more than three voxels have *P* > 0 for all elements in the kernel, the center voxel should be set to zero (this is substantial in the 3 × 3 kernel). In contrast, if the larger kernel is used, more spatial blurring is likely to be introduced. In ^23^Na‐MRI, GM and WM are in general the tissues of interest. Thus, the CSF region in the regression mask is excluded so that only two unknowns need to be estimated by the regression. A general overview of the 2D LR approach is shown in Supplementary Figure 1. However, prior to excluding the CSF region, the contributions of high TSC in CSF to GM and/or WM, and signal cross‐contamination between GM and WM (ie spill‐over effects), should be considered. This can be corrected for by convolving the PSF with a tissue mask followed by subtracting each smoothed tissue mask signal from the original sodium image (see Figure 1). Note that the PVC was performed on native sodium space after anatomical information was mapped onto the sodium data using boundary‐based registration. In this work, we used a Gaussian PSF with FWHM of 1.21 pixels (4.8 mm).^19^ The method is similar to that of Muller‐Gartner et al in PET,^20^ but the difference arises because the TSC of WM is not necessarily homogeneous in ^23^Na‐MRI. For example, the correction of spill‐in effects from CSF and WM can be described with the following equation: *S*_GM:spillin‐corr_ = *S*_orig_ − (*S*_WM_ ⊗PSF × *P*_WM_ ⊗PSF + *S*_CSF_ ⊗PSF × *P*_CSF_ ⊗PSF), where *S*_GM:spillin‐corr_ is the spill‐in effect‐corrected TSC for the GM, *S*_orig/GM/WM_ is the bias‐field corrected sodium signal, and the symbol ⊗ is the convolution operation. The reasons for smoothing measured sodium signals with PSF prior to subtracting them from original sodium data were the following: (i) we did not assume homogeneous WM and CSF signal in sodium data, and (ii) it enabled the method to be more robust to the low SNR of sodium MR data and inhomogeneous signal distribution over the brain in the high‐field scanner.

In order to reduce inherent spatial blurring in the kernel regression, we propose the use of 3D‐mLTS. This approach not only effectively removes outliers by computing the residuals of each data point, but also retains sufficient information by use of an *n* × *n* × *n* kernel. The 3D kernel itself can also facilitate the reduction of spatial blurring, as the radius of the kernel is reduced whilst retaining the number of voxels. For example, the number of voxels for a 5 × 5 kernel using the conventional 2D LR method is approximately matched to those for a 3 × 3 × 3 kernel in our proposed method. Typically, a 5 × 5 × 5 kernel induces excessive spatial blurring, especially for small lesion volumes (data not shown), and thus we excluded it from quantitative comparison. The 2D‐mLTS method has been previously described for PVC of ASL MRI.^17^ Briefly, an initial estimation of regression parameters is obtained using a sorting‐based method, assuming the intensity of each voxel to be similar to that of the central voxel in the kernel. After calculating the residuals for all voxels (eg 3 × 3 × 3 voxels in this study) and sorting in ascending order, the new subset is created based on the trimming parameter, *α*. The number of voxels used in the subsequent regression is determined by multiplying *α* by the kernel size. The regression parameters are then estimated from the new subset again. Ten iterations are performed for the procedures of calculating residuals and the parameter estimations from the new subset, as convergence was found to occur after 10 iterations.

### 2.2 ^23^Na‐MRI simulations

The simulated dataset was generated from a TPM obtained by segmenting a 3D high‐resolution T1w image of one healthy volunteer. The TPM was registered and down‐sampled to the native ^23^Na‐MRI space of the subject. To create a pseudo‐TSC map for this simulation, we assigned the published TSC values to binary masks generated for each tissue type (GM, 48 mmol/L; WM, 43 mmol/L).^21^ In addition, two spherical lesions (radius 10 and 15 mm) with different TSC values (70 and 30 mM) were added to the simulated dataset in order to assess the degree of spatial blurring. The generated pseudo‐TSC map was converted to a PVE‐contaminated TSC map by multiplying by the TPM registered to ^23^Na‐MRI. The PVE‐contaminated TSC map was employed to compare the performance of PVC methods for conventional 2D and 3D LR, and the 3D‐mLTS. The simulations were performed using two kinds of dataset, one without adding random noise and the others with the addition of random noise of different magnitudes (ie white noise of amplitude equal to 3 and 6) to simulate a more realistic sodium MR image. Monte Carlo simulations were conducted on the simulated data with random noise for 100 iterations to evaluate the effects of SNR on the performance of the 3D‐mLTS method. Note that the purpose of the simulation was to evaluate the performance of correction for tissue fraction effects rather than spill‐over effects. Thus, we did not take the PSF into account in this simulation.

#### 2.2.1 Phantom

Details for the phantom experiments are provided in the Supplementary Materials.

### 2.3 MR image acquisitions

Eight healthy volunteers were recruited for our study (age 20‐35 years, male = 8) and written informed consent was obtained from all subjects. All experimental protocols were approved by the Institutional Review Board (IRB) of Gachon University Gil Medical Center. The MRI experiments were conducted with a whole‐body 7 T MR system (Siemens Healthcare, Erlangen, Germany) using a custom‐built eight‐channel dual‐tuned ^23^Na/^1^H head coil, which consists of two looped coils. The outer loop (8 × 14 cm) was tuned for transmission and receipt of ^1^H (297.2 MHz) signals, while the inner loop (7 × 13 cm) was tuned for transmission and receipt of sodium (78.61 MHz) signals. Prior to the ^23^Na‐MRI acquisition, RF pulse power for each subject was calibrated empirically to account for differences in coil loading. For the calibration, we used a non‐selective free induction decay sequence with the following parameters: *T*_R_/*T*_E_ = 25 000/0.5 ms, bandwidth = 3 kHz, RF pulse shape sinc, pulse duration = 1 ms, vector size = 1024. RF pulses were tested at variable power and the attenuator was then calibrated for a 90° pulse, when a maximum value was obtained. For *B*_0_ inhomogeneity correction, the shimming was performed using the water ^1^H signal prior to acquiring the sodium image. The vendor‐provided 3D field map was employed to achieve the optimal signal over the entire volume. For *B*_1_ bias field correction, we obtained the coil sensitivity map for each subject by extracting the central portion of the *k*‐space data. The sodium MR images were acquired using a modified 3D fast and low angle shot (FLASH) sequence with the following parameters: *T*_R_/*T*_E_ = 100/4 ms, flip angle = 90°, excitation pulse sinc with 1 ms duration, field of view (FOV) = 384 × 384, matrix size = 96 × 96, slice thickness = 4 mm, number of slices = 48, number of averages = 6, pixel bandwidth = 260 Hz, orientation of slice sagittal, scan time = 49 min 48 s. Anatomical information was obtained with a T1w 3D magnetization prepared rapid acquisition GRE (MPRAGE) sequence with the following parameters: *T*_R_/*T*_E_ = 2140/4.82 ms, inversion time = 1190 ms, FOV = 256 × 256, slice thickness = 1 mm, flip angle = 10°, 1 mm isotropic resolution, number of averages = 1, scan time = 7 min 41 s. Image reconstruction and the combination of each coil image was performed offline using a custom written MATLAB script (MathWorks, Natick, MA).

### 2.4 MR image analysis

One subject was excluded from further analysis due to a large motion artifact. A flowchart depicting the overall processing pipeline is shown in Figure 1. Briefly, the T1w images were corrected for intensity non‐uniformity and subsequently adaptive non‐local mean (ANLM) filtered in order to deal with spatially‐varying noise levels in the MR images.^22^ The parameters used in ANLM filtering are the size of the search and similarity windows, which were set to 7 × 7 × 7 and 3 × 3 × 3, respectively. The TPMs for GM, WM, and CSF of the individual T1w data were obtained by segmenting ALNM‐filtered T1w images using SPM12 software. (https://www.fil.ion.ucl.ac.uk/spm/software/spm12). Each brain tissue mask was generated from the TPM by converting the tissue probability to a binary value. Rather than applying a threshold value to TPM for the generation of each tissue mask, we compared tissue compositions in every single voxel and then selected the maximum composition as corresponding tissue. We further manually segmented the CSF mask into ventricle and sulci CSF in order to evaluate the differences in TSC values estimated in accordance with reference to different CSF compartments. The ALNM‐filtered T1w image was then co‐registered to the sodium image using FSL FLIRT with a boundary‐based registration cost function,^23^, ^24^ and an affine transformation matrix was applied to register the TPM in T1w space into native ^23^Na‐MRI. Prior to applying the PVC method, the sodium MR data was first corrected for the bias field using the sodium coil sensitivity map followed by correcting for spill‐over effects using a Gaussian PSF with a FWHM of 4.8 mm (Figure 1). The 3D‐mLTS method used to correct the tissue fraction effect was applied in the native sodium space with a kernel size of 3 × 3 × 3 with a trimming parameter *α* = 0.4 (see Section 3 for the determination). We also performed the other PVC methods (conventional 2D and 3D LR) to quantitatively compare performance in vivo. The 3D‐mLTS method was applied separately for GM and WM to account for signal cross‐contamination. Finally, an in vivo TSC map was obtained using the ventricle signal as a reference, with the assumption that the sodium concentration of the ventricle is 138 mM.^21^

### 2.5 Evaluation of in vivo PVC performance

For individual in vivo ^23^Na‐MRI data, we estimated the regression errors as calculated by the square root of the mean squared error (RMSE):

(3) $$\text{RMSE}=\sqrt{\frac{{\left(\bar{S}-P\cdot \bar{m}\right)}^{T}\cdot \left(\bar{S}-P\cdot \bar{m}\right)}{d.f.}}$$

where d.f. is the degrees of freedom, being (*n* ^2^ − 2) and (*n* ^3^ − 2) for regression of the 2D and 3D LR method, respectively. The RMSE was used for in vivo evaluation of the performance of the different PVC methods as no ground truth data is available.

### 2.6 Statistical analysis

The RMSE values from the individual in vivo ^23^Na‐MRI data were compared between the different PVC methods. One‐way analysis of variance (ANOVA) followed by Bonferroni's post hoc test was conducted to evaluate whether the RMSEs were statistically different between the PVC methods employed. *P*‐values less than 0.05 were considered statistically significant. All statistical analyses were performed using MATLAB Statistical Toolbox (MathWorks).

## 3 RESULTS

### 3.1 Simulations and phantom

**FIGURE 2:**
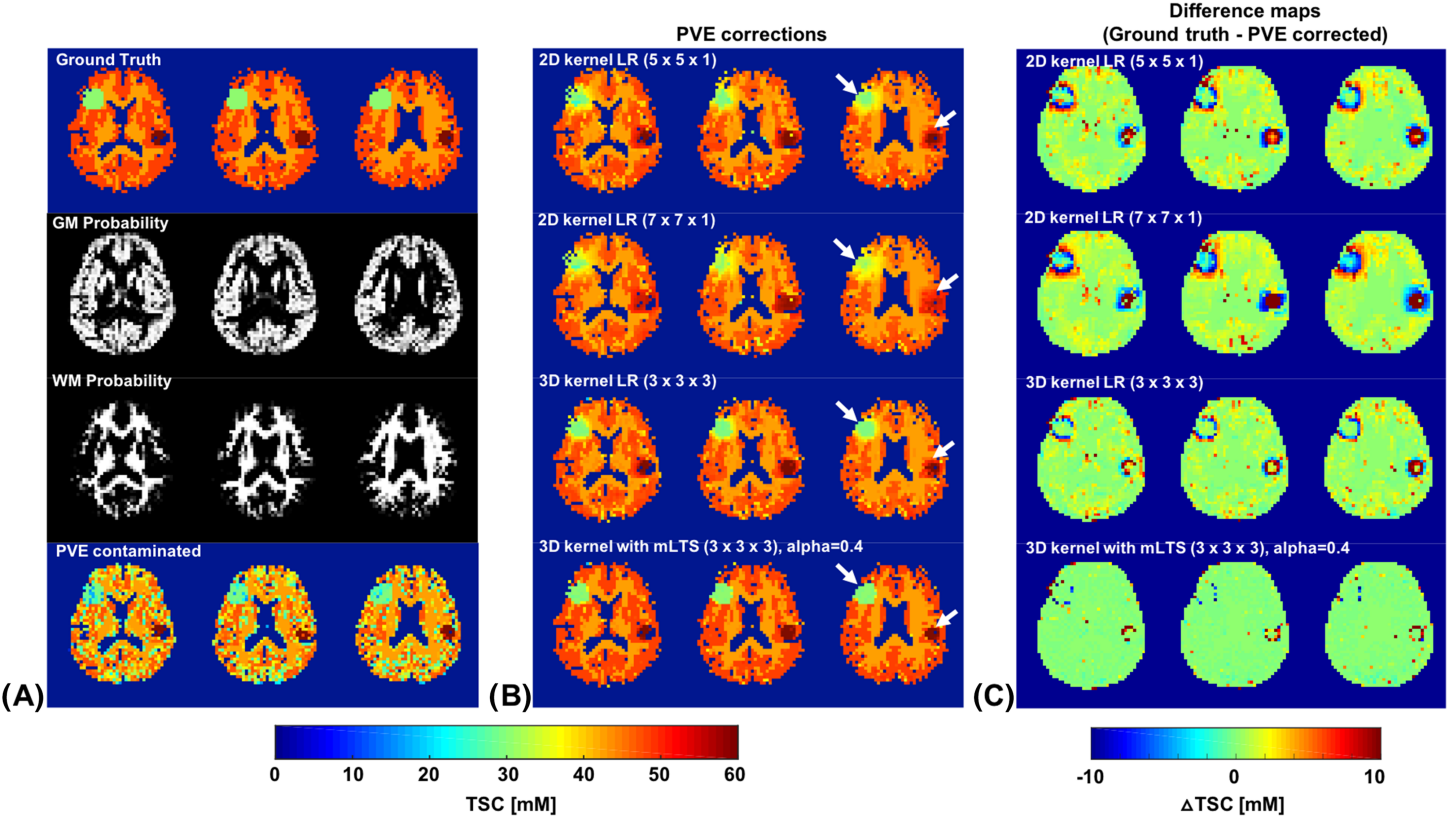
Simulation results for PVCs of noise‐free data. A, A ground truth image (top row) was generated by assigning published TSC values as GM (48 mM) or WM (43 mM). To assess the extent of spatial blurring, two spherical lesions (eg radius 10 mm and 15 mm) were included to mimic hyper‐TSC (70 mM) and hypo‐TSC (30 mM). The TPMs for GM and WM (second and third rows) were obtained by segmenting a high‐resolution T1w image from a representative subject. A PVE‐contaminated image was then produced by multiplying the ground truth by the TPMs. B, PVC results for different kernel sizes of the LR and 3D‐mLTS methods. White arrows indicate the extent of spatial blurring in the focal lesions. C, Difference between ground truth and PVE‐corrected data, provided for easier comparison of the performance of the PVCs. Note that the simulations only consider the tissue fraction effects, and not spatial blurring effects, due to the broad PSF

Prior to the quantitative comparison of the different PVC methods, we first simulated the effects of different *α* values on the performance of the 3D‐mLTS method to select the optimal trimming parameter (Supplementary Figure 2). By visual inspection, the performances of the 3D‐mLTS method in the range of *α* values between 0.3 and 0.7 appeared not to be significantly different. By calculating the mean of the absolute differences between ground truth and PVE‐corrected data for each *α* value, the *α* value of 0.4 was selected as optimal for further analysis. We then compared the performances of different PVC methods for the simulations without adding random noise. Figure 2A shows the simulated ^23^Na‐MRI data for ground truth (top row), TPMs for GM and WM (second and third rows), and PVE‐contaminated data (fourth row). Figure 2B shows PVE‐corrected data for different kinds of PVC method: conventional 2D LR with 5 × 5 (top row) and 7 × 7 kernels (second row), 3D LR with a 3 × 3 × 3 kernel (third row), and 3D‐mLTS with *α* = 0.4 (fourth row). Without correcting for the tissue fraction effects, the TSCs of GM and WM in the simulated PVE‐corrupted data were underestimated by 20% and 11%, respectively. Furthermore, it produces poor contrast between GM and WM. Similarly, the hyper‐ and hypo‐TSCs of the lesions in the PVE‐corrupted data were also underestimated by 24.5% and 15.6%, respectively. After applying voxel‐wise PVC methods, the underestimated TSC values were recovered toward ground truth data. It can be clearly seen that the 3D‐mLTS method yielded superior results in terms of spatial blurring, indicated by white arrows. The differences between ground truth and PVE‐corrected data using the 3D‐mLTS method were only approximately 0.6% and 0.4% for GM and WM, and 9.7% and 6.3% for hyper‐ and hypo‐TSC lesions, respectively. The relatively small lesion size (10 mm radius) for hyper‐TSC was attributed to a larger error. As expected, the 2D kernel with a larger size (7 × 7) induced the greatest spatial blurring, which significantly underestimated the true TSC value in the lesion masks. The performance of the different kinds of PVC method can be seen in the difference map shown in Figure 2C. The simulation results are summarized in Table 1.

**TABLE 1:** Mean TSCs for regions of interest in the simulations without the addition of random noise (unit: mM)

	Ground truth	Uncorrected	PVC methods			
			LR 5 × 5	LR 7 × 7	LR 3 × 3 × 3	3D‐mLTS
GM	48	38.5	46.9	46.5	46.9	47.7
WM	43	38.1	42.1	41.9	42.1	42.8
Hyper‐TSC lesion	70	52.8	57.7	53.7	60.4	63.2
Hypo‐TSC lesion	30	25.3	33.6	35.5	32.9	31.9

**FIGURE 3:**
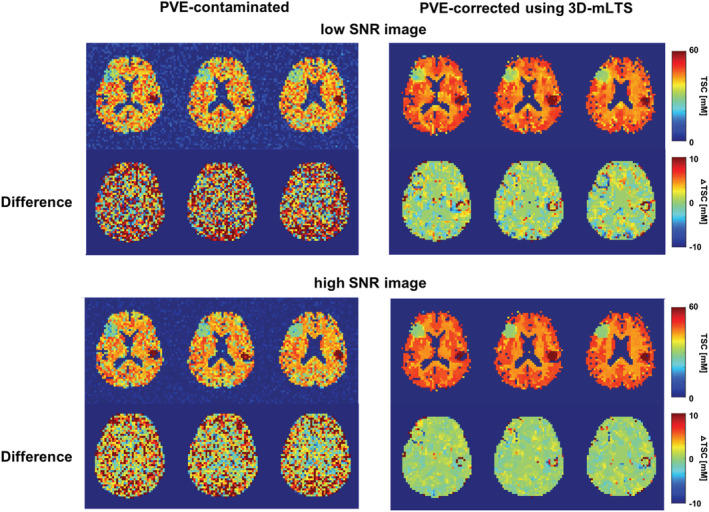
Monte Carlo simulation results for the effects of SNR on performance of the 3D‐mLTS method. Two different magnitudes of Gaussian random noise were added to the PVE‐contaminated data. The same ground truth data as shown in Figure 2 was used for the simulation. A, The images shown in the first row are the PVE‐contaminated and corrected TSC maps in the simulation for low SNR, whereas the second row shows the difference in maps between ground truth and PVE‐contaminated (left) or corrected data (right). The yellow to red colors represent the underestimation of TSC while light‐blue to blue colors represent overestimations in the difference maps. B, The same as A, but with the simulation performed on a high‐SNR image. Note that the difference maps clearly demonstrate the underestimations of TSC when not considering tissue fraction effects. Even after applying PVC using the 3D‐mLTS method, the edges of the lesions still represent imperfect signal restorations, particularly for low‐SNR images

Monte Carlo simulations were further performed to investigate the effects of SNR on the performance of the 3D‐mLTS method. Two different magnitudes of Gaussian random noise to mimic low‐ and high‐SNR images were applied to simulated PVE‐corrupted data. As shown in Figure 3, the 3D‐mLTS method performed better in the high‐SNR image than in the low‐SNR image. To further highlight the tissue fraction effects in low‐resolution ^23^Na‐MRI, the difference maps between ground truth and PVE‐corrupted data are also shown, with Table 2 summarizing the results.

**TABLE 2:** Monte Carlo simulation results for the differences in TSCs (*∆*TSCs) between ground truth and tissue fraction‐corrected (or uncorrected) low‐ and high‐SNR sodium images. The tissue fraction effects were corrected for using the 3D‐mLTS method. Note that error magnitudes are larger in hyper‐TSC lesions than hypo‐TSC lesions, due to the small lesion sizes

	*∆*TSCs (mM), mean (SD)			
	Low SNR		High SNR	
	Uncorrected	Tissue fraction corrected	Uncorrected	Tissue fraction corrected
GM	10.97 (0.03)	4.04 (0.03)	9.80 (0.01)	3.13 (0.03)
WM	7.13 (0.05)	1.76 (0.03)	5.59 (0.02)	0.82 (0.01)
Hyper‐TSC lesion	10.86 (0.33)	8.12 (0.35)	9.79 (0.20)	7.01 (0.18)
Hypo‐TSC lesion	5.52 (0.18)	3.39 (0.29)	3.93 (0.11)	2.36 (0.11)

The NaCl phantom experiment clearly shows blurring of the sodium MR image at the edge of small tubes (Supplementary Figure 3) due to both PSF‐induced spill‐over and tissue fraction effects. Even when the thickness of the tube is larger than 4 mm (Tubes 11 and 12), there are still overlapping signal intensities at the edges of the tubes.

### 3.2 In vivo evaluation of PVC methods in ^23^Na‐MRI

**FIGURE 4:**
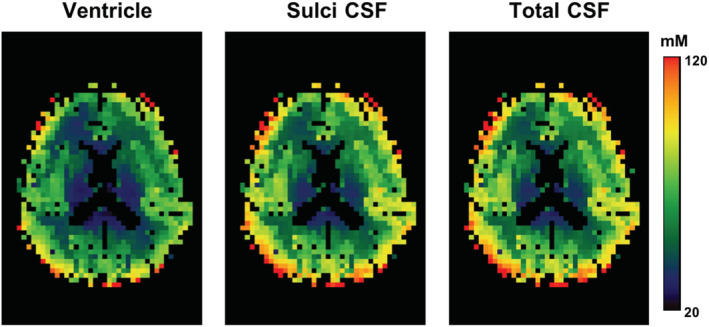
In vivo TSC maps of a slice from a representative subject after applying the 3D‐mLTS method. However, the TSCs were calculated using different CSF compartments as references (left, ventricle; middle, sulci CSF; right, total CSF (ventricle + sulci CSF))

**FIGURE 5:**
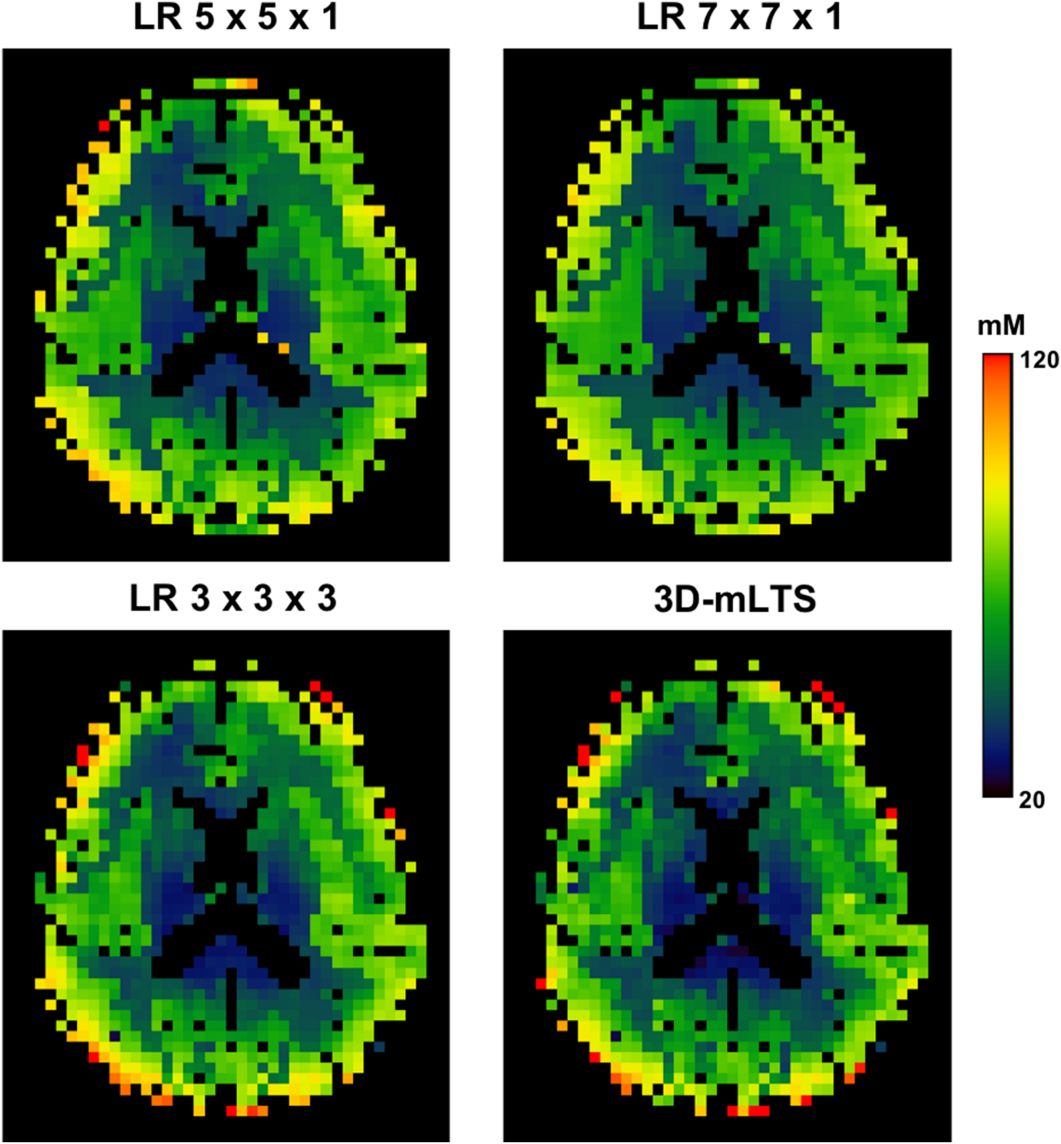
Effects of various PVC methods on TSC estimation in vivo. The conventional 2D LR approach with a 7 × 7 kernel results in the most blurring, while the 3D‐mLTS method preserves more spatial details with greater anatomical contrast

**FIGURE 6:**
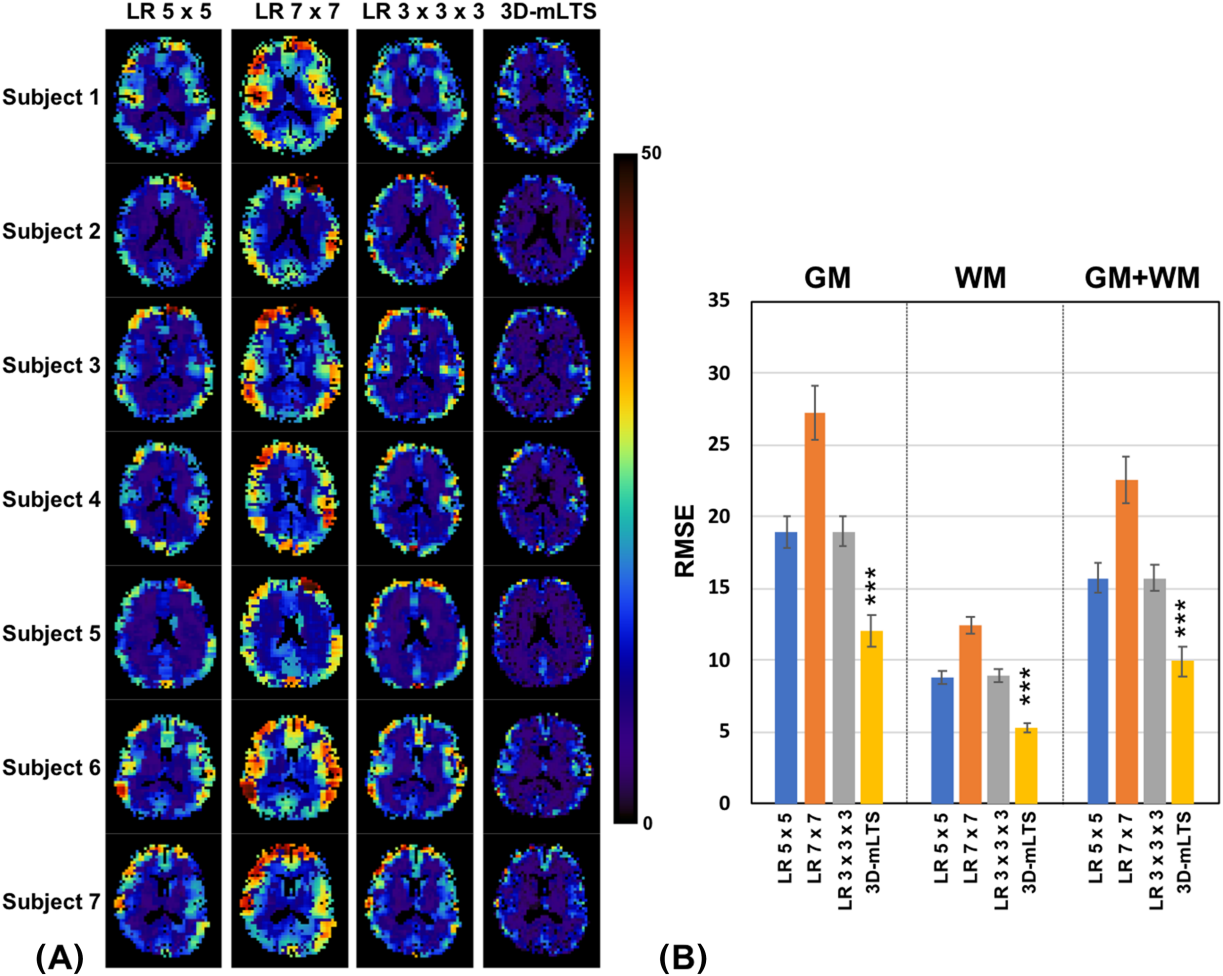
A, RMSE maps calculated from each of the PVC methods using a slice from each subject. B, Bar graph showing the mean RMSE values across all GM, WM, and GM + WM voxels. Error bars indicate standard deviation. Significance level: ***p < 0.001 (details of the statistical methods are provided in Section 2)

**FIGURE 7:**
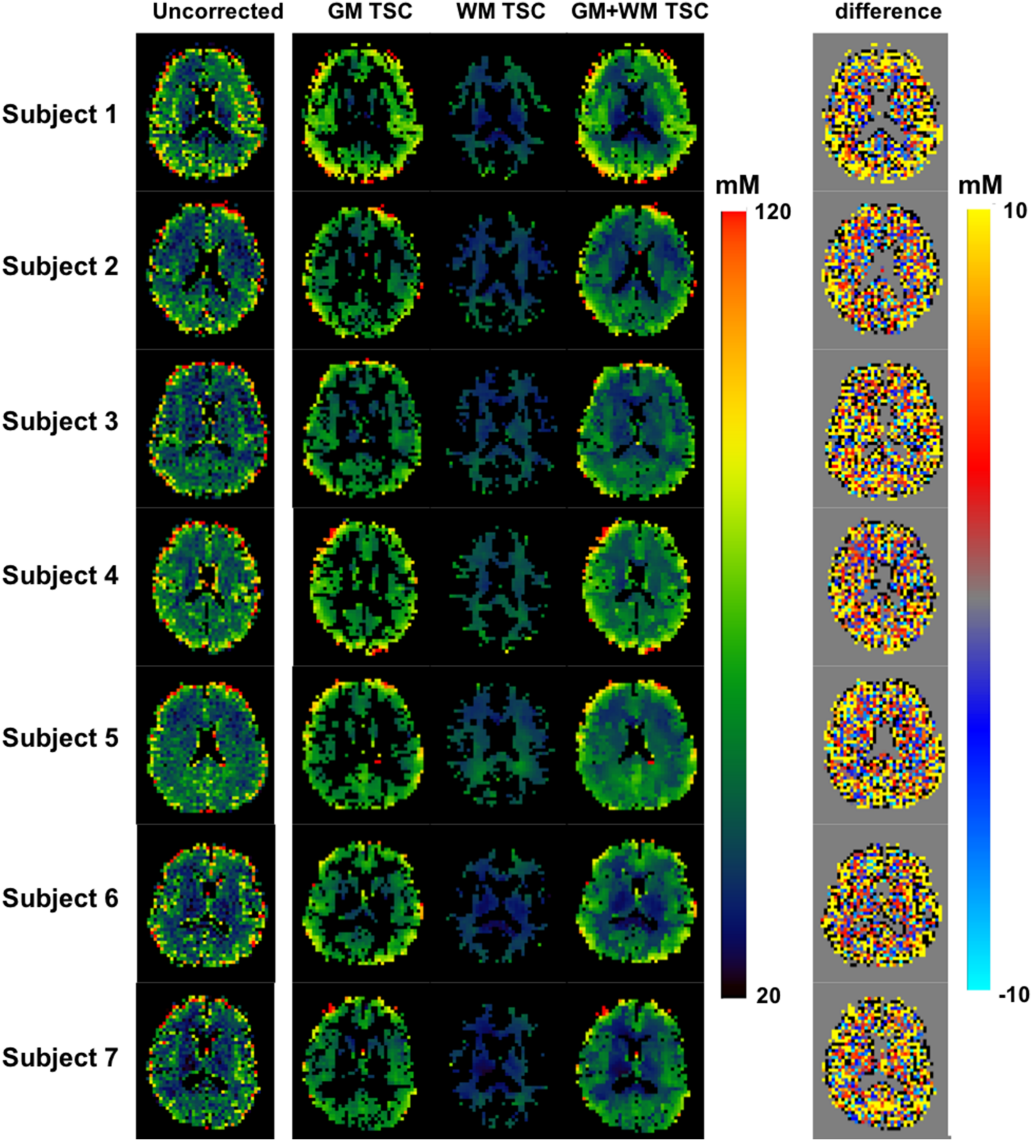
In vivo TSC maps of a representative slice after applying 3D‐mLTS for each subject. The TSCs were calculated using ventricle signals as the reference. Uncorrected ^23^Na‐MRI data are shown in the first column for comparison. For visualization purposes, the TSC maps for GM and WM are displayed separately. Differences between uncorrected and PVE‐corrected data are also shown. Note that the spill‐over effects were corrected for prior to applying the voxel‐wise 3D‐mLTS method

Figure 4 shows the in vivo TSC maps after correcting the PVEs (both spill‐over and tissue fraction effects) for a slice of a representative subject, but the TSC values were calculated using different CSF compartments for the comparison. It is obvious that the sodium signals in ventricle and sulci CSF were quite different (ie sulci CSF < ventricle), resulting in discrepant TSCs in the GM and WM of the brain. Figure 5 shows the same in vivo TSC maps as shown in Figure 4, but different PVC methods were employed to compare the performance of each PVC method. Clearly, the conventional LR approach with a 7 × 7 kernel produced the most blurred TSC map, while the proposed 3D‐mLTS method preserved more spatial details. Mean TSC values in GM and WM of each subject before and after correcting for PVEs are summarized in Table 3. It should be noted that the TSCs of GM and WM in the uncorrected data were overestimated by 18% and 27% as compared with those in spill‐over‐corrected data, whereas TSCs of GM and WM in spill‐over‐corrected data were underestimated by 7% and 5%, respectively, as compared with those in tissue‐fraction‐corrected data using the 3D‐mLTS method (regardless of reference regions). For the quantitative comparisons of each PVC method, RMSE maps for all subjects are provided in Figure 6, demonstrating that the 3D‐mLTS method resulted in the lowest RMSE values as compared with the other methods. Note that the 3D‐mLTS method reduces the regression errors in the brain edge areas (Figure 6A). Statistical results for mean RMSE values in the GM and WM masks of each individual sodium MR image are shown in Figure 6B. One‐way ANOVA revealed that there were significant differences in the RMSEs of GM (*F*(3, 27) = 152.73, *p* < 0.001), WM (*F*(3, 27) = 260.9, *p* < 0.001) and GM + WM (*F*(3, 27) = 134.58, *p* < 0.001) between the PVC methods. Bonferroni's post hoc test showed that the RMSEs for the 3D‐mLTS method were significantly lower than those for the other PVC methods (*p* < 0.001), but the RMSEs for conventional LR with a 5 × 5 kernel were not significantly different from those for the LR with a 3 × 3 × 3 kernel (*p* > 0.05). Figure 7 shows the in vivo TSC maps estimated using ventricle signals as the reference in a representative slice of each individual dataset. For easy comparison before and after correcting for PVEs (ie both spill‐over and tissue fraction effects), uncorrected ^23^Na‐MRI data are also shown in the first column. Note that there are greater anatomical contrasts between GM and WM after applying the 3D‐mLTS method, as the outputs for kernel regression approach are the weighed local tissue fractions. The difference maps between uncorrected and PVE‐corrected data indicate the underestimation (red‐yellow color) and overestimation (blue‐light blue and black colors) of the TSC values.

**TABLE 3:** TSCs of each subject before and after PVC. The TSCs for GM and WM with reference to different CSF compartments are provided for comparison

No	Uncorrected						Spill‐over corrected						PVE corrected†					
	Ventricle		Sulci CSF		Total CSF		Ventricle		Sulci CSF		Total CSF		Ventricle		Sulci CSF		Total CSF	
	GM	WM	GM	WM	GM	WM	GM	WM	GM	WM	GMM	WM	GM	WM	GM	WM	GM	WM
1	66.33	43.96	77.40	51.31	78.66	52.14	56.09	34.41	65.46	40.16	66.53	40.82	63.80	36.46	74.43	42.55	75.66	43.25
2	66.55	43.53	78.80	51.55	80.26	52.51	55.72	33.73	65.98	39.94	67.20	40.68	61.25	35.48	72.51	42.01	73.96	42.80
3	58.83	40.41	79.81	54.82	80.87	55.54	49.39	32.12	67.00	43.57	67.89	44.14	53.18	33.77	72.13	45.81	73.02	46.41
4	69.45	48.67	77.29	54.16	79.05	55.40	59.75	38.32	66.50	42.64	68.01	43.61	63.70	40.11	70.81	44.64	72.52	45.65
5	62.13	44.62	80.74	57.99	81.15	58.28	54.17	35.91	70.41	46.68	70.76	46.91	58.07	38.10	75.48	49.52	75.87	49.77
6	64.04	39.89	72.09	44.90	73.50	45.79	54.27	31.11	61.09	35.01	62.29	35.70	57.41	32.69	64.60	36.80	65.86	37.52
7	60.66	39.46	75.01	48.80	75.63	49.20	50.96	31.13	63.02	38.49	63.54	38.81	53.64	32.68	66.32	40.41	66.92	40.74
Mean	64.00	42.93	77.31	51.93	78.44	52.69	54.34	33.82	65.64	40.93	66.60	41.53	58.72	35.61	70.90	43.11	71.97	43.73
SD	3.71	3.28	2.96	4.27	2.86	4.21	3.42	2.66	2.97	3.77	2.86	3.71	4.39	2.82	4.04	4.06	4.02	3.99
CoV	5.79	7.64	3.82	8.22	3.64	7.99	6.29	7.86	4.52	9.21	4.29	8.93	7.47	7.91	5.69	9.41	5.58	9.12

^†^ PVEs include both spill‐over effects resulting from PSF and tissue fraction effects due to the low spatial resolution of ^23^Na‐MRI. CoV, coefficient of variation (%) defined as standard deviation divided by mean.

## 4 DISCUSSION

We have demonstrated a robust voxel‐wise PVC method (ie 3D‐mLTS—correcting tissue fraction effects) in ^23^Na‐MRI with an attempt to reduce the inherent spatial blurring for the accurate determination of TSC values in the brain. This has further highlighted the necessity of correcting for the tissue fraction effects in relatively low‐spatial‐resolution ^23^Na‐MRI. Importantly, two major sources of the PVEs, the tissue fraction and signal spill‐over effects, were considered for the accurate estimation of in vivo TSCs. The sources of PVEs were clearly demonstrated in NaCl phantom experiment, which emphasizes again the importance of the PVC in ^23^Na‐MRI. This is the first study to show the voxel‐wise PVC results for GM and WM in ^23^Na‐MRI and the method was rigorously validated in the simulation and in vivo data. The simulations and in vivo evaluation of PVC methods revealed that 3D‐mLTS outperforms the other PVC methods (eg conventional 2D and 3D LR) in terms of *∆*TSC between ground truth and PVE‐corrected data, and the RMSEs, which were computed using Equation 3 for the regression error involving in vivo assessment of the PVC methods. Quantitative evaluation of the PVC methods using both simulated and in vivo data demonstrated that *∆*TSC and RMSE values increased with the kernel size. This might be due to the violation of local uniformity for the larger kernel.^16^ Nonetheless, the larger kernel can have more equations available for the regression, thereby avoiding the possibility of rank deficiency, but it would have greater spatial smoothing effects after the PVC. Therefore, a trade‐off exists for the selection of kernel size, which should be optimized. A major difference between our proposed method (3D‐mLTS) and those developed for other imaging modalities lies in the use of a 3D kernel combined with the mLTS method, which not only reduces spatial blurring but also increases the accuracy of TSC estimation. The degree of inherent spatial blurring induced by the regression algorithm was further evaluated by employing two spherical lesions with different TSCs in the simulations (Figure 2). This is especially important when the assessment of small areas with increased TSC is needed, such as cases involving tumors, stroke, and multiple sclerosis.

To the best of our knowledge, there is only one previous report referring to the PVC method in ^23^Na‐MRI, where the GTM method was employed.^21^ The GTM method was originally developed for PET PVC. A drawback of the GTM method is that it does not generate a PVE‐corrected image, as the approach is based on parcellated brain region‐wise correction. Unlike voxel‐based PVC methods, GTM PVC attempts to correct for the effects of PSF causing signal smearing between neighboring regions. However, it is important to note that the TSC values estimated after the PVC in this work were higher in the GM and lower in the WM as compared with those reported using the GTM method. The discrepancy in the TSCs might be explained by the different acquisition schemes (*T*_E_, 4 ms versus 0.35 ms; *k*‐space sampling, Cartesian versus radial) and/or the correction methods for the PVEs. Another popular method for PVC is the Muller‐Gartner algorithm, which was originally formulated to remove spill‐in from the WM and CSF compartments as well as spill‐out from the GM compartment. Unlike GTM, the Muller‐Gartner method provides voxel‐wise results and considers the tissue fraction effects. However, the tissue of interest in the Muller‐Gartner method is only for GM, with the assumption that the WM signals are constant across all WM voxels and the contribution from the CSF is zero. The assumptions are not valid for ^23^Na‐MRI studies where contamination from high TSCs in the CSF is present and the TSCs in the WM are not necessarily homogeneous across all WM voxels. In addition, a threshold value for the probability map of the GM must be chosen to obtain the tissue‐fraction‐corrected GM signals. Thus, the results of the PVC using the Muller‐Gartner method depend on the threshold value to prevent noise amplification by dividing by a number close to 0. In this work, the proposed 3D‐mLTS method to correct for the tissue fraction effects can exclude such a possibility, as it is based on a general linear model that represents the voxel intensity as a weighted sum of each tissue contribution. It is worth noting, however, that the higher *∆*TSC between ground truth and PVE‐corrected data was observed in the smaller spherical lesion even after applying the 3D‐mLTS method (ie underestimation of true concentration by ~10%) as demonstrated by our simulations (Figures 2 and 3). This issue has been addressed in a previous report, highlighting a large underestimation of sodium signal in small‐volume lesions and the limitation of low‐resolution ^23^Na‐MRI.^25^ The improvement of spatial resolution in ^23^Na‐MRI would resolve this concern.

The most striking finding of the present study was that the spatial blurring and regression errors in correction of the PVEs can be minimized by the use of a 3D kernel combined with an mLTS algorithm, as shown with both the simulated and in vivo data. The use of a 3D kernel alone instead of a conventional 2D kernel can also reduce to some extent the spatial smoothing effect (Figure 2) and regression error. The reason is that the 3D kernel radius is smaller than the 2D whilst preserving total voxel numbers, which consequently leads to less in‐plane spatial blurring. Importantly, the 3D kernel itself does not impose any additional computational load compared with the conventional 2D LR method. However, it should be noted that there might be greater through‐plane spatial blurring in the case of using only a 3D kernel for the PVC. The idea to reduce the effects of through‐plane smoothing in the current study originates from LTS regression, which is based on the subset whose least square fit has the smallest sum of squared residuals.^18^ Indeed, we found that the extent of spatial blurring was dramatically reduced in the lesion volumes and approximately 40‐50% reductions in RMSEs were obtained with the use of the 3D‐mLTS method when compared with the 2D or 3D kernel LR method (Figure 7).

Given the importance of PVC in studying aging and neurodegenerative diseases (as signal cross‐contamination from nearby regions is a confounding factor when determining whether a difference in the TSC across time or between groups is due to difference in tissue properties or atrophy‐induced bias), the proposed PVC method enables accurate quantification of TSC while minimizing PVE‐induced bias in pathological circumstances. Furthermore, a precise estimation of TSC renders the interpretation of results biologically plausible. In recent times, the application of ^23^Na‐MRI in clinical studies has become increasingly popular due to appreciation of the pivotal roles of sodium in regulating metabolic integrity and ion homeostasis.^26^

Despite evidence for superior PVC performance of the 3D‐mLTS method, our study has some limitations that should be considered. First, we were not able to perform an external reference measurement with known sodium concentrations, which could be directly used for simple linear calibration to calculate the TSCs of the brain in vivo. The reason for the lack of phantom measurement calibration was that the custom‐built ^1^H/^23^Na coil was tightly fitted with most of the subjects during imaging, which made placing the phantom within the FOV of image acquisition difficult. Thus, we estimated the TSCs using ventricle signal as a reference. Most in vivo studies of brain ^23^Na‐MRI provide TSC values for GM and WM with^21^ or without considering the PVE,^10^, ^11^, ^12^, ^27^, ^28^ which falls in the ranges of 30‐70 mM for GM and 20‐60 mM for WM. The estimated in vivo TSCs in this work are also within the ranges, supporting the validity of our method. Second, the sodium *T*_2_ relaxation time in tissue is very short with biexponential characteristics. The rapid decay of the short *T*_2_ component causes a signal loss of up to 60%, resulting in a low SNR for ^23^Na‐MRI. The echo time used in this study was 4 ms, which might raise concerns about *T*_2_ signal loss. Nonetheless, the reason for the use of Cartesian sampling rather than radial acquisition with a short echo time was to reduce the spatial blurring effects caused by the PSF, as the FWHM of PSF in the radial acquisition is broader than that of the Cartesian equivalent, which has been addressed in a previous report.^19^ Furthermore, the sequence used in this work can be readily available on most clinical scanners, thereby expanding the utility of ^23^Na‐MRI studies for diagnostic purposes. We believe that future studies using an ultrashort *T*_E_ sequence with corrections for both tissue fraction and spill‐over effects would enable more accurate estimation of TSCs in vivo. Third, we did not consider the potential difference in the PSF between ^23^Na‐ and ^1^H‐MRI. We believe that correcting for the tissue fraction effects by utilizing TPM generated by segmenting a high‐resolution T1w image is less likely to be affected by the PSF difference between them. Instead this might be strongly dependent on the registration accuracy. We achieved highly reliable registration results using the boundary‐based registration algorithm. Finally, the PSF used for correcting spill‐over effect in this study was a Gaussian shape even though the PSF in Fourier transform MRI has a sinc‐like shape that can lead to a so‐called Gibbs ringing effect in images acquired with very small matrix size. Moreover, the shape of the PSF is determined by the *k*‐space sampling method and the number of phase‐encoding steps applied. The Gibbs ringing effect is particularly problematic in spectroscopic application (eg MRSI) where spatial resolution is lower than that of ^23^Na‐MRI and the leakage from lipid‐rich voxels (eg subcutaneous region) can contaminate ^1^H spectra. However, it should be noted that the Gibbs ringing effect induced by a sinc‐shaped PSF may be neglected when more than 64 phase‐encoding steps are employed.^29^ In our work, we used 96 phase‐encoding steps to acquire ^23^Na‐MRI data. From this point of view, we found that the PSF approximation with Gaussian shape used in this study did not make much difference in the result for correction of CSF spill‐in effect when compared with that obtained with a sinc‐shaped PSF (data not shown).

In conclusion, we have described a voxel‐wise PVC method for the accurate estimation of TSCs considering both the spill‐over and tissue fraction effects in ^23^Na‐MRI at 7 T, and demonstrated its validity in terms of restoring under‐ or overestimated TSCs as well as regression error. Our findings demonstrated that the estimated TSCs in the brain can be strongly biased when PVEs are not considered, suggesting the necessity of PVC in low‐resolution ^23^Na‐MRI. The 3D‐mLTS method therefore appears to be well suited for the accurate determination of TSCs, especially for focal lesions common in pathological conditions.

## CONFLICTS OF INTEREST

The authors declare that the research was conducted in the absence of any commercial or financial relationships that could be construed as a potential conflict of interest.

## Supporting information


**Table S1.** The diameters of each tube of phantom and corresponding NaCl concentrations
**Figure S1.** The simple illustration for kernel‐based linear regression (LR) approach. In this example, 2D 3 × 3 kernel is shown. We want to estimate true signal intensity of GM and WM for the voxel S_22_, which is weighted sum of GM and WM signal where weighting coefficients are tissue probability. The linear regression model (right top equation) has two unknowns in one equation available. By assuming that true signal intensities over the 3 × 3 kernel is constant, we can bring more equations to solve the equation. The solution can be found by matrix inversion.
**Figure S2.** The optimization of trimming parameter, α in the 3D‐mLTS method. The simulations were conducted with different α values in range of 0.3 to 0.7. For each α value, the difference maps (*∆*TSC) between ground truth and PVEs‐corrected data were calculated. We found that the mean *∆*TSC over the brain tissue mask was minimal at the α values of 0.4.
**Figure S3.** The schematic diagram for constructed sodium phantom is shown in (A). The six tubes (#3 ‐ #6, #9 and #10) are used for calibration and measured signal intensities for the 6 tubes are plotted as function of the known NaCl concentration (B). The estimated NaCl concentration map using calibration curve is shown. Note that partial volume effects (PVEs) are visible at small diameter tubes (#13 and #14), due to the point spread function (PSF) as well as tissue fraction effects (blurred boundary between the tubes (#11 and #13, and #12 and #14).
