# Supplementary material for: Voxel‐wise partial volume correction method for accurate estimation of tissue sodium concentration in 23Na‐MRI at 7 T
Source: NMR Biomed. 2020 Dec 3;34(2):e4448. doi: 10.1002/nbm.4448 (PMC7816248; doi:10.1002/nbm.4448)
Supplement: Supplementary file 1 — Table S1. The diameters of each tube of phantom and corresponding NaCl concentrations Figure S1. The simple illustration for kernel‐based linear regression (LR) approach. In this example, 2D 3 × 3 kernel is shown. We want to estimate true signal intensity of GM and WM for the voxel S22, which is weighted sum of GM and WM signal where weighting coefficients are tissue probability. The linear regression model (right top equation) has two unknowns in one equation available. By assuming that true signal intensities over the 3 × 3 kernel is constant, we can bring more equations to solve the equation. The solution can be found by matrix inversion. Figure S2. The optimization of trimming parameter, α in the 3D‐mLTS method. The simulations were conducted with different α values in range of 0.3 to 0.7. For each α value, the difference maps (∆TSC) between ground truth and PVEs‐corrected data were calculated. We found that the mean ∆TSC over the brain tissue mask was minimal at the α values of 0.4. Figure S3. The schematic diagram for constructed sodium phantom is shown in (A). The six tubes (#3 ‐ #6, #9 and #10) are used for calibration and measured signal intensities for the 6 tubes are plotted as function of the known NaCl concentration (B). The estimated NaCl concentration map using calibration curve is shown. Note that partial volume effects (PVEs) are visible at small diameter tubes (#13 and #14), due to the point spread function (PSF) as well as tissue fraction effects (blurred boundary between the tubes (#11 and #13, and #12 and #14). [file NBM-34-e4448-s001.docx]

**Supplementary Information**

**Supplementary Methods and Results**

***Phantom***

We constructed a phantom consisting of 14 tubes with different diameters filled with different sodium chloride (NaCl) concentrations (25, 50, 75, 100, 125 and 150 mM). The information of the diameters and the NaCl concentrations for each tube is provided in Supplementary Table 1 (Table S1). The overall volume of the phantom was approximately 1.7 L. The six tubes (#3 - #6, #9 and #10) are used for calibration while the other 8 tubes are used to examine the extent of spatial blurring caused by the PSF of Cartesian sampling in ^23^Na-MRI acquisition. For 23Na-MRI measurement, the experiment was conducted using the same acquisition protocol with *in vivo* measurement, but the number of averages were reduced to 4. The MPRAGE data for the phantom was also acquired with the same protocol with in vivo measurement. Each tube mask was manually segmented on high-resolution T1w data and the tube masks were downsampled to sodium phantom data. The NaCl concentrations for each tube were estimated through calibration curve, which was obtained by simple linear relationship between measured signal intensity of each tube and known concentration.

The schematic diagram and estimated NaCl map for the phantom are shown in Supplementary Figure 3 (Figure S3). As shown in Figure S3-(C), there are visible PVEs especially for small diameter of tubes (#13 and #14) even though we acquired the sodium data with Cartesian sampling. The blurred boundary between the tubes are most likely due to both the PSF and tissue fraction effects.

**Table S1.** The diameters of each tube of phantom and corresponding NaCl concentrations

| # Phantom | Inner (mm) | Outer (mm) | NaCl [mM] |
| --- | --- | --- | --- |
| 1 | 39.4 | 45.6 | 75 |
| 2 | 39.4 | 45.6 | 125 |
| 3 | 18.8 | 25.2 | 125 |
| 4 | 18.8 | 25.2 | 25 |
| 5 | 14 | 18.2 | 75 |
| 6 | 14 | 18.2 | 150 |
| 7 | 14 | 18.2 | 25 |
| 8 | 14 | 18.2 | 150 |
| 9 | 18.8 | 25.2 | 50 |
| 10 | 18.8 | 25.2 | 100 |
| 11 | 30 | 40.4 | 50 |
| 12 | 30 | 40.4 | 100 |
| 13 | 9.54 | 13.54 | 75 |
| 14 | 14 | 18.2 | 50 |
| surrounding | 150 | 160 | 40 |

**Supplementary Figures**

**Figure S1.** The simple illustration for kernel-based linear regression (LR) approach. In this example, 2D 3 × 3 kernel is shown. We want to estimate true signal intensity of GM and WM for the voxel S_22_, which is weighted sum of GM and WM signal where weighting coefficients are tissue probability. The linear regression model (right top equation) has two unknowns in one equation available. By assuming that true signal intensities over the 3 × 3 kernel is constant, we can bring more equations to solve the equation. The solution can be found by matrix inversion.


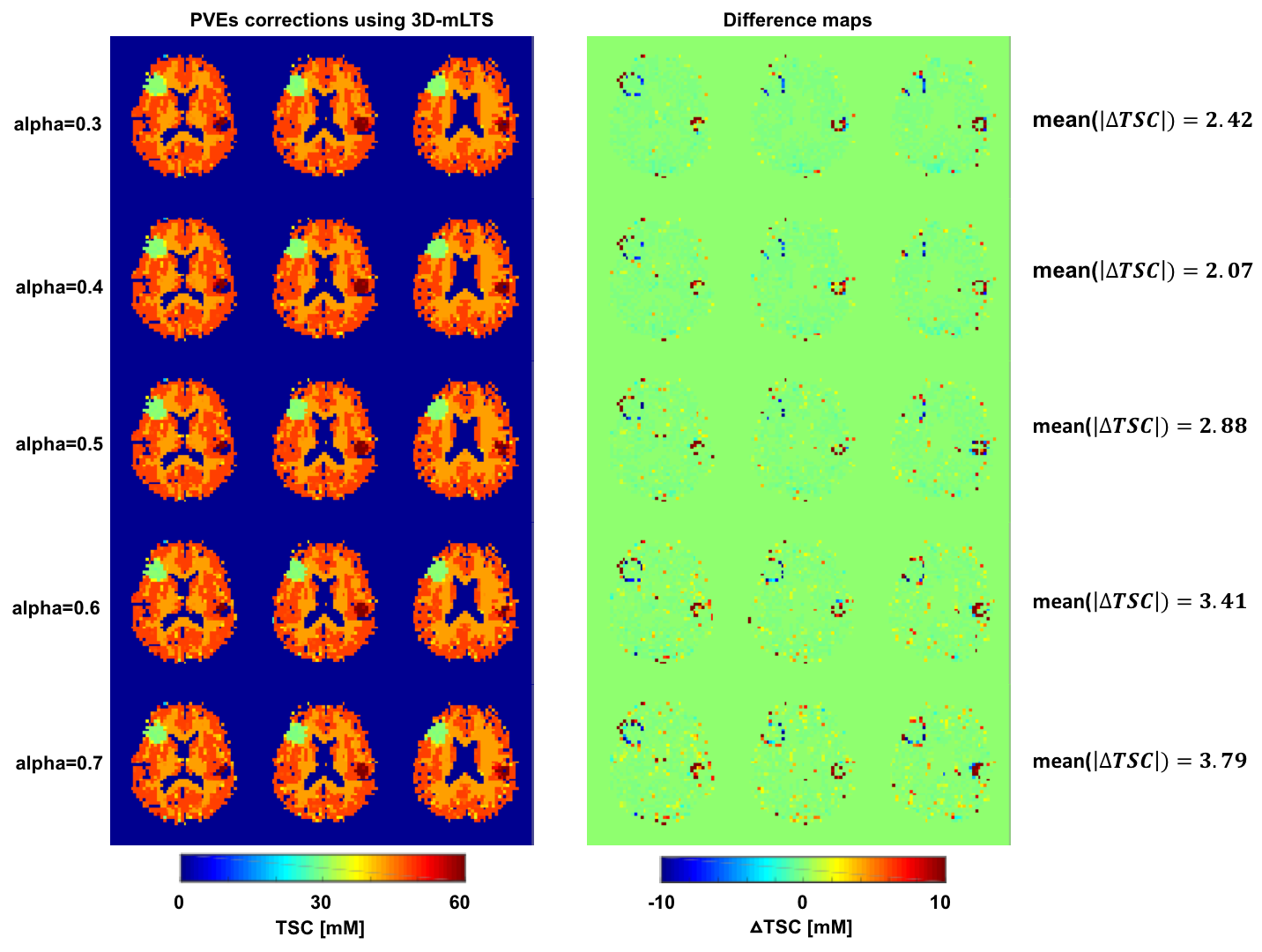


**Figure S2.** The optimization of trimming parameter, α in the 3D-mLTS method. The simulations were conducted with different α values in range of 0.3 to 0.7. For each α value, the difference maps ($\Delta$TSC) between ground truth and PVEs-corrected data were calculated. We found that the mean $\Delta$TSC over the brain tissue mask was minimal at the α values of 0.4.

**
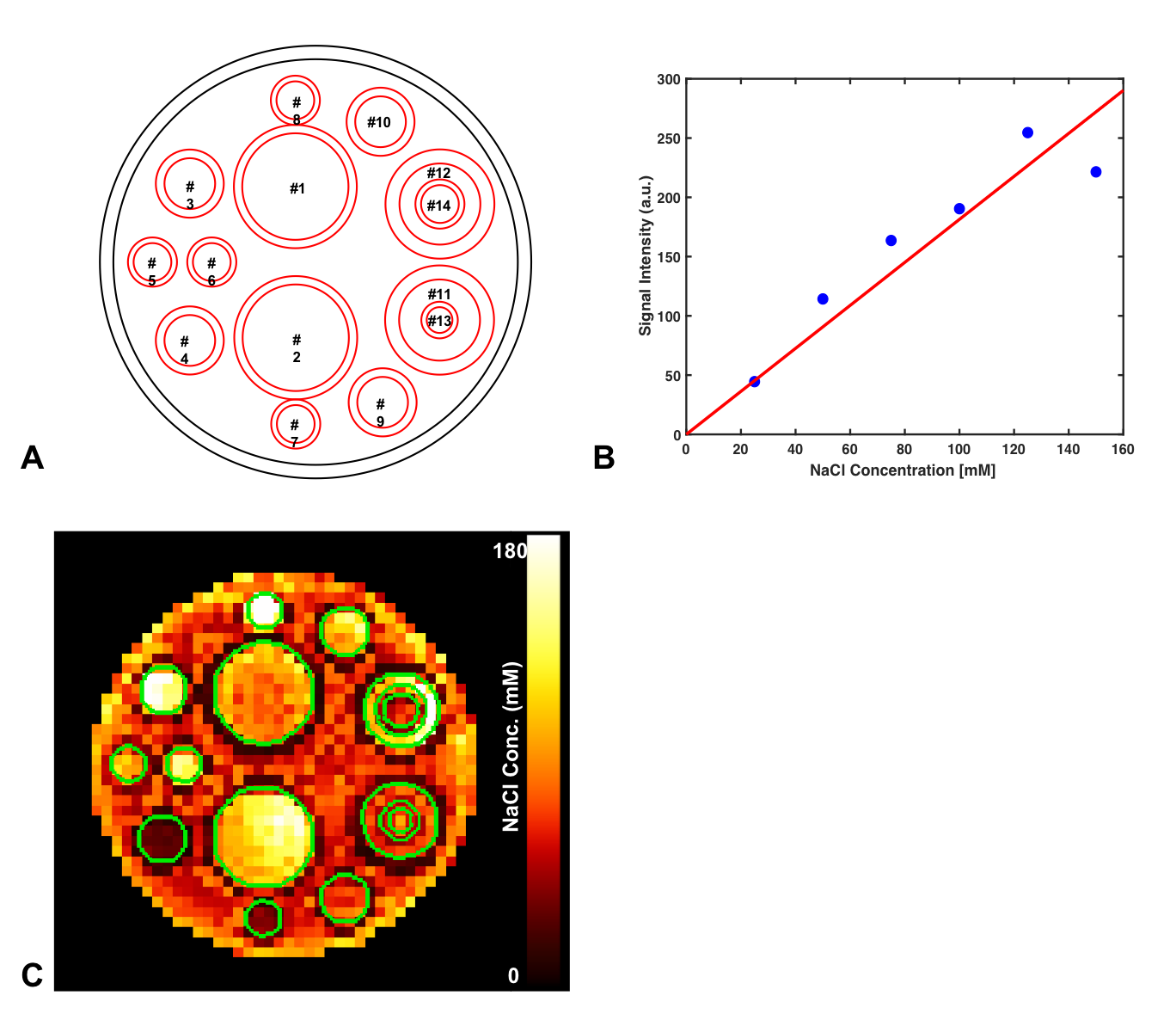
**

**Figure S3.** The schematic diagram for constructed sodium phantom is shown in (A). The six tubes (#3 - #6, #9 and #10) are used for calibration and measured signal intensities for the 6 tubes are plotted as function of the known NaCl concentration (B). The estimated NaCl concentration map using calibration curve is shown. Note that partial volume effects (PVEs) are visible at small diameter tubes (#13 and #14), due to the point spread function (PSF) as well as tissue fraction effects (blurred boundary between the tubes (#11 and #13, and #12 and #14).
